# The effect of switchgrass loadings on feedstock solubilization and biofuel production by *Clostridium thermocellum*

**DOI:** 10.1186/s13068-017-0917-7

**Published:** 2017-11-30

**Authors:** Tobin J. Verbeke, Gabriela M. Garcia, James G. Elkins

**Affiliations:** 1 BioEnergy Science Center, Oak Ridge National Laboratory, Oak Ridge, TN 37831-6038 USA; 2 Biosciences Division, Oak Ridge National Laboratory, Oak Ridge, TN 37831-6038 USA; 3 Present Address: Department of Biological Sciences, University of Calgary, Calgary, AB T2N 1N4 Canada

**Keywords:** *Clostridium thermocellum*, Consolidated bioprocessing, Switchgrass, Recalcitrance, Inhibition, High-solid loading, Ethanol

## Abstract

**Background:**

Efficient deconstruction and bioconversion of solids at high mass loadings is necessary to produce industrially relevant titers of biofuels from lignocellulosic biomass. To date, only a few studies have investigated the effect of solids loadings on microorganisms of interest for consolidated bioprocessing. Here, the effects that various switchgrass loadings have on *Clostridium thermocellum* solubilization and bioconversion are investigated.

**Results:**

*Clostridium thermocellum* was grown for 10 days on 10, 25, or 50 g/L switchgrass or Avicel at equivalent glucan loadings. Avicel was completely consumed at all loadings, but total cellulose solubilization decreased from 63 to 37% as switchgrass loadings increased from 10 to 50 g/L. Washed, spent switchgrass could be additionally hydrolyzed and fermented in second-round fermentations suggesting that access to fermentable substrates was not the limiting factor at higher feedstock loadings. Results from fermentations on Avicel or cellobiose using culture medium supplemented with 50% spent fermentation broth demonstrated that compounds present in the supernatants from the 25 or 50 g/L switchgrass loadings were the most inhibitory to continued fermentation.

**Conclusions:**

Recalcitrance alone cannot fully account for differences in solubilization and end-product formation between switchgrass and Avicel at increased substrate loadings. Experiments aimed at separating metabolic inhibition from inhibition of hydrolysis suggest that *C. thermocellum*’s hydrolytic machinery is more vulnerable to inhibition from switchgrass-derived compounds than its fermentative metabolism.

## Background

Efficient plant cell-wall deconstruction and solubilization is a major challenge to overcome when converting lignocellulosic feedstocks to renewable fuels and chemicals. One promising low-cost strategy to produce cellulosic ethanol through bioconversion is consolidated bioprocessing (CBP), which relies on the simultaneous solubilization and fermentation of lignocellulose carbohydrate polymers without additional enzymes [1]. The hydrolytic capabilities of the thermophile, *Clostridium* (*Ruminiclostridium*) *thermocellum* have identified this bacterium as a particularly capable organism for CBP [2, 3]. In addition, genetic engineering efforts have improved the bacterium’s abilities to detoxify pretreatment derived inhibitors [4] as well as to achieve high ethanol yields and titers simultaneously [1, 5].

Differences in feedstock type and composition [6–8], time of harvest [3, 9] and pretreatment strategies [10, 11] have all been previously assessed in regard to *C. thermocellum*-mediated conversion to ethanol. However, little has been reported regarding the effect that substrate loading has on *C. thermocellum* solubilization and biofuel production despite the realization that feedstock loadings in excess of > 100 g/L carbohydrate are considered essential for industrialization and economic viability of cellulosic ethanol [12, 13]. Furthermore, studies that have looked at differences in substrate loadings have typically employed model cellulosic substrates or soluble cellodextrins and have principally focused on end-product distribution profiles [1, 12, 14, 15].

High-solid fermentations of real-world biomass are known to produce a variety of challenges to biocatalysts. For example, soluble sugar accumulation [16, 17], reductions in enzyme adsorption [18], and end-product induced cellulase inactivation [19] have all been reported to adversely affect solubilization by systems employing fungal enzymes. Only a few studies investigating solids loadings on CBP-candidate microbes have been reported to date, however. Using *Clostridium phytofermentans*, decreased sugar conversion efficiencies were observed as loadings of washed, pretreated corn stover increased [20]. The reduction in conversion efficiency observed was attributed to an accumulation of the fermentation product acetate, which was proposed to principally inhibit the strain’s solubilization machinery, rather than its ability to metabolically ferment the saccharides. Amongst CBP-relevant thermophiles, *Caldicellulosiruptor bescii* has been reported to grow on unpretreated switchgrass at concentrations as high as 200 g/L [21, 22]. Furthermore, solubilization efficiencies (27–33%) remained consistent for the bacterium at biomass loadings ranging from 1 to 50 g/L switchgrass with improved overall conversions achievable through biomass washing and repetitive fermentations. It was, however, unclear why individual fermentations stopped at ~ 30% solubilization, though an unidentified inhibitor associated with spent fermentation broths was noted [21].

The recalcitrance barrier is one that all bioconversion strategies face, though the magnitude of this barrier is known to vary widely [3]. Similarly, the processes affected by high-solid loading induced inhibition can also vary depending on the feedstock, process configuration, and biocatalyst. The intent of this study is to provide an initial assessment of the effects that varied biomass loadings of “minimally-pretreated” (autoclaved) switchgrass have on *C. thermocellum*’s solubilization and conversion capabilities. It further seeks to provide insight into what processes are most vulnerable to inhibition at increased loadings.

## Results

**Fig. 1:**
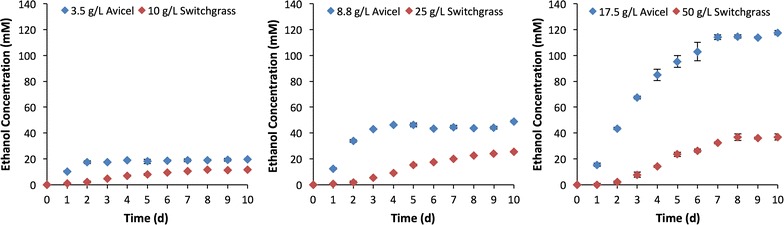
Net ethanol production by *C. thermocellum* M1570 under various substrate loadings. For all graphs, the glucan content in the Avicel fermentations is equivalent to those in the switchgrass fermentations at the corresponding loading. Values are averages of triplicate fermentations and error bars represent standard deviation

Batch fermentations of minimally pretreated switchgrass or Avicel were run in parallel to compare solubilization and biofuel production by *C. thermocellum*. The glucan contents were normalized between comparator fermentations based on a reported glucan content of 35% cellulose in the Alamo cultivar [22–24]. At 3.5, 8.8, and 17.5 g/L of Avicel, the ethanol yields for *C. thermocellum* M1570 ranged from 50 to 60% of the theoretical maximum, which is consistent with previous reports for the strain [25]. In the switchgrass fermentations, however, there was a significant drop in the overall ethanol titer (Fig. 1). At 10, 25, and 50 g/L loadings, ethanol titers decreased by 41, 48, and 69%, respectively, relative to those observed in the corresponding Avicel fermentations. Mass-balance analyses confirmed that the increased switchgrass loadings affected ethanol production, but also decreased total fermentation end-products by 21, 33, and 59% in the 10, 25, and 50 g/L switchgrass loadings, respectively (Table 1).

**Table 1:** Mass-balance analyses of Avicel and switchgrass fermentations

Condition	Initial substrate/ biomass (mg)	Residual substrate/biomass + cell dry weight (mg)	Soluble glucose and xylose equivalents recovered (mg)	Fermentation products recovered (mg)^a^	Carbon recovery (%)	Final pH
3.5 g/L Avicel	175.0	13.8 ± 1.1	5.0 ± 0.1	90.4 ± 2.9	62.4 ± 2.8	7.0 ± 0.0
8.8 g/L Avicel	440.0	30.7 ± 0.6	4.1 ± 0.3	225.1 ± 10.6	59.1 ± 2.8	6.8 ± 0.0
17.5 g/L Avicel	875.0	44.9 ± 1.3	1.5 ± 0.1	548.0 ± 6.1	67.9 ± 2.4	6.0 ± 0.0
10 g/L SG	500.0	297.9 ± 12.0	124.1 ± 16.2	71.0 ± 7.3	98.6 ± 8.9	7.1 ± 0.0
25 g/L SG	1250.0	811.1 ± 7.7	179.2 ± 14.0	150.8 ± 2.2	91.3 ± 3.1	6.8 ± 0.0
50 g/L SG	2500.0	1768.8 ± 10.7	285.6 ± 11.3	223.3 ± 11.6	91.1 ± 2.9	6.6 ± 0.0

Values are averages (*n* = 3) ± SD

*SG* switchgrass

^a^Sum total of net acetate, lactate, formate, ethanol, and CO_2_ production. CO_2_ was estimated based on the formula: CO_2_ = acetate + ethanol—formate

Near complete glucan utilization was observed in the Avicel fermentations (Table 1). Five-to-eight percent of the initial substrate mass was recovered in the cell pellet fraction after 10 days of fermentation, which is consistent with the expected amounts of biomass produced by *C. thermocellum* growth [26, 27]. Only minor amounts of glucose equivalents were observed in the remaining supernatant fraction. This was in stark contrast to the switchgrass fermentations, where significant quantities of soluble sugars were recovered. For the switchgrass fermentations, 39, 53, and 97 mg of glucose equivalents, as well as 85, 127, and 189 mg of xylose equivalents, were recovered in the 10, 25, and 50 g/L switchgrass loadings, respectively (Table 1). Together, these account for 25, 14, and 11% of the initial biomass provided in the 10, 25, and 50 g/L conditions. A mass balance accounting for fermented and soluble residual glucans showed that 63, 47, and 37% of the total glucose equivalents were removed from the initial 10, 25, and 50 g/L switchgrass loadings, respectively (Table 2).

**Table 2:** Cellulose solubilization efficiencies under different switchgrass loadings

	10 g/L	25 g/L	50 g/L
Solubilized glucans^a^ (mg)	110.2	203.4	320.2
Initial glucan (mg)	175.0	437.5	875.0
Solubilization efficiency (%)	63.0	46.5	36.6

^a^Sum of fermentation end-products and solubilized, but non-fermented glucose equivalents

The effective solubilization and fermentation of 17.5 g/L Avicel by *C. thermocellum* suggested that the basis for inhibition in the switchgrass experiments was not related to end-product inhibition, nutrient availability, or pH limitation (Table 1). Additional experiments were then designed to determine the contribution that biomass recalcitrance, metabolic inhibition, and/or inhibition of hydrolysis contributed to the observed reduction in end-products formed.

**Fig. 2:**
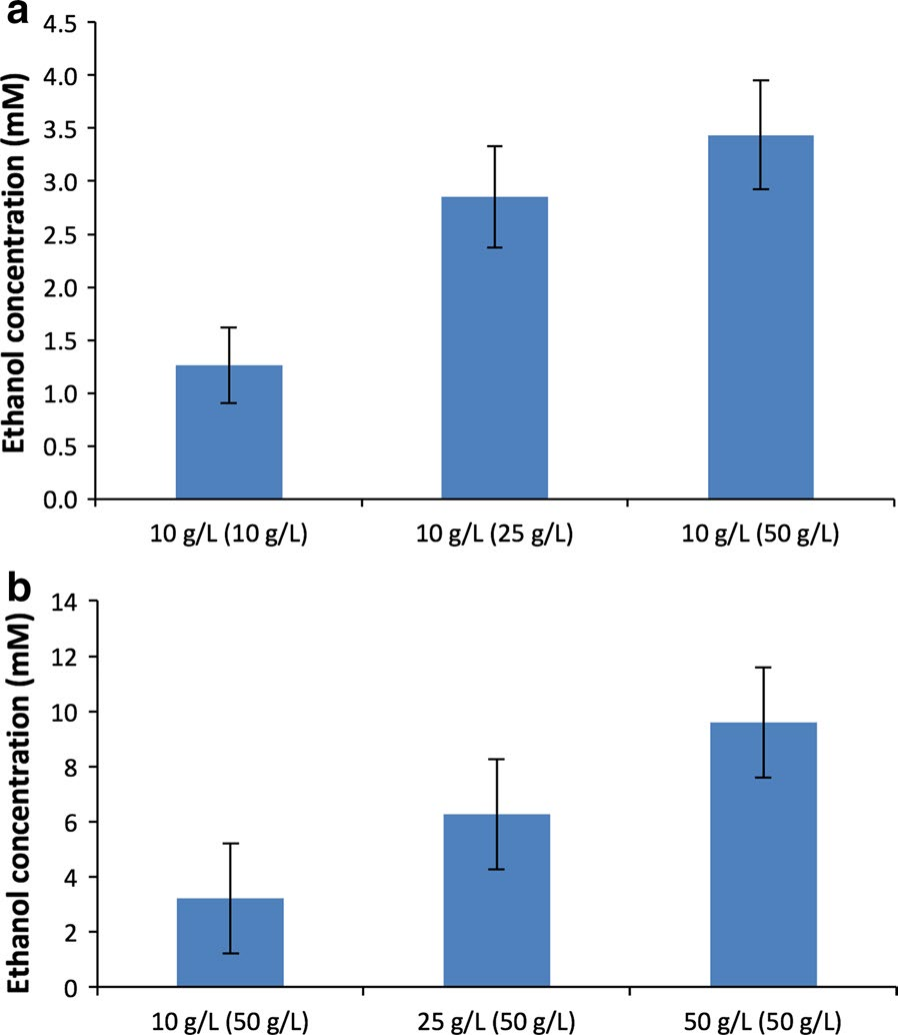
Ethanol production by *C. thermocellum* M1570 during second-round fermentations of switchgrass. **a** Ethanol production on 10 g/L washed biomass from the initial 10, 25, and 50 g/L switchgrass fermentations. **b** Ethanol production at different loadings using the original 50 g/L switchgrass after washing. All values are averages (*n* = 6) from two independent experiments. Error bars represent standard deviation

To examine the contribution of recalcitrance, washed residual switchgrass recovered from the initial experiments was subjected to a second round of fermentation using fresh growth medium and 10 g/L of the spent switchgrass. At equivalent solids loadings, the highest ethanol titer was observed in switchgrass recovered from the initial 50 g/L fermentation (Fig. 2a). In terms of efficiency, *C. thermocellum* was able to solubilize and ferment an additional 13, 24, and 23% of the remaining glucan equivalents after the initial 10, 25, and 50 g/L switchgrass fermentations, respectively.

Varying levels of inhibition were observed based on differences in feedstock loading in the primary fermentations. By extension, residual glucan content of the spent feedstock would also then vary and be dependent on loading conditions. To account for variability in residual glucan content due to differences in first-round solubilization, an additional set of secondary fermentation experiments were conducted using residual switchgrass from the initial 50 g/L loading only. As expected, the ethanol titers after a second 10-day fermentation were the greatest at the highest biomass loading (Fig. 2b). Despite the higher titers, however, the efficiency of sugar conversion to end-products once again decreased as biomass loading increased. Specifically, at second-round loadings of 10, 25, or 50 g/L switchgrass, *C. thermocellum* solubilized and fermented an additional 22, 17, and 13% of the residual glucan.

Based on the hydrolysis and end-product formation profiles observed in the second-round fermentations, recalcitrance alone could not: (i) fully account for the differences in end-product titers observed in the initial switchgrass and Avicel fermentations or (ii) explain why ethanol titers plateaued during the first-round fermentations when glucans were still available for solubilization and conversion (Fig. 1). The potential for switchgrass-derived compounds to inhibit *C. thermocellum* metabolism was then assessed. Culture broths comprised of 50% fresh growth medium and 50% neutralized, spent broth from the initial fermentation were used. Cellobiose was provided as a soluble cellodextrin at a glucan loading equivalent to 17.5 g/L Avicel. Under all conditions tested, > 95% of all available glucose equivalents provided were consumed (Table 3). Furthermore, significant ethanol production was observed with end-product ratios remaining relatively consistent across all conditions. The sole exception was the significant decrease in both ethanol and formate production in cultures containing 50% supernatant derived from the original 17.5 g/L Avicel fermentations. These lower titers were observed despite similar consumption of cellobiose relative to the other samples as well as the control. The reductions in titer represent changes in net production from cellobiose and do not account for residual end-products from the initial fermentations. For example, cultures with supernatant derived from the initial 17.5 g/L Avicel fermentations only produced an additional 91.0 ± 5.8 mM of ethanol (Table 3), but the actual ethanol concentration in the fermentation medium was 150 ± 6.1 mM when accounting for ethanol produced in the first-round fermentations.

**Table 3:** Net end-product formation of cellobiose^a^ fermentations containing 50% (v/v) of spent supernatant

Condition	Concentration (mM)						Final pH
	Ethanol	Formate	Acetate	Lactate	CO_2_	Glucose equivalents remaining	
Control	116.5 ± 5.5	11.7 ± 0.9	4.0 ± 0.6	0.7 ± 0.3	108.8 ± 9.9	1.9 ± 0.5	6.2 ± 0.0
3.5 g/L Avicel	110.7 ± 4.1	9.8 ± 0.6	3.2 ± 0.3	0.4 ± 0.0	104.1 ± 6.6	1.4 ± 0.1	6.2 ± 0.0
8.8 g/L Avicel	100.1 ± 1.8	9.3 ± 1.2	3.5 ± 0.2	0.6 ± 0.3	94.3 ± 8.4	3.0 ± 0.4	6.1 ± 0.0
17.5 g/L Avicel	91.0 ± 5.8	7.6 ± 1.0	4.1 ± 0.8	0.1 ± 0.1	87.5 ± 11.3	3.9 ± 0.4	6.0 ± 0.0
10 g/L SG	101.6 ± 2.8	11.5 ± 1.7	6.5 ± 1.0	0.7 ± 0.2	96.6 ± 9.2	1.8 ± 0.7	6.1 ± 0.0
25 g/L SG	101.4 ± 1.3	11.8 ± 0.6	4.8 ± 0.4	0.3 ± 0.0	94.4 ± 8.3	2.4 ± 0.1	6.1 ± 0.1
50 g/L SG	107.1 ± 3.2	9.4 ± 0.9	5.5 ± 0.2	0.4 ± 0.0	103.2 ± 5.5	4.6 ± 0.4	6.0 ± 0.0

*SG* switchgrass

^a^Fermentations contained cellobiose at a concentration of 97 mM glucose equivalents. *n* = 6

**Fig. 3:**
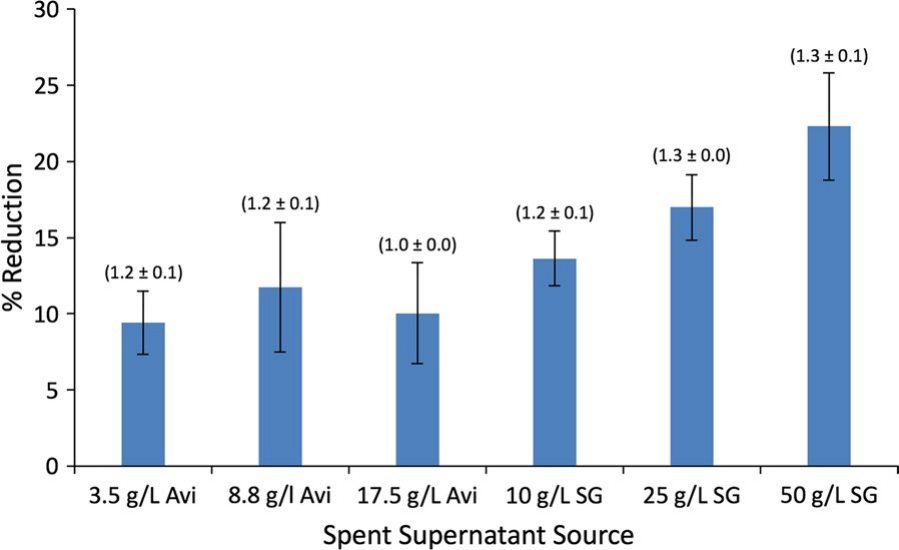
Reduction in the solubilization efficiencies of 17.5 g/L Avicel in fermentations containing 50% (v/v) spent supernatant. *X*-axis labels indicate the source of the spent supernatant from the first-round fermentation conditions used. Values in brackets above columns represent the molar ethanol production ratios (mM ethanol produced: mM glucose equivalents consumed). *Avi* Avicel, *SG* switchgrass

Next, inhibition of hydrolysis was examined using fermentation medium containing 50% spent broth (as above), but using 17.5 g/L Avicel instead of cellobiose. The molar ethanol yields remained relatively consistent across all conditions with one exception (Fig. 3). Once again, the fermentation broth comprised of 50% spent supernatant from the original 17.5 g/L Avicel fermentation showed less ethanol production than the other conditions. Specifically, a reduction in ethanol yield was observed (Fig. 3) that was similar to the reduction in titer determined from the cellobiose fermentations (Table 3).

Unlike the cellobiose fermentations, however, there was significant variation in the glucose equivalents remaining. Total Avicel solubilization was less for cultures containing supernatant from the initial switchgrass fermentations relative to those containing supernatant from the initial Avicel fermentations. The greatest reduction in solubilization was observed in cultures containing supernatant from the initial 50 g/L switchgrass fermentations. Here, ~ 22% less Avicel was hydrolyzed after 10 days of incubation than was observed in the control condition.

## Discussion

The plant cell-wall solubilization efficacy of *C. thermocellum* has been well established with glucan utilization efficiencies up to 60–70% on multiple potential bioenergy crops, including switchgrass [3, 9, 28]. Recalcitrance alone, however, cannot fully account for the differences in fermentation end-product titers observed here between the Avicel and switchgrass fermentations (Fig. 1, Table 1). If recalcitrance was the sole factor, it would be expected that total solubilization and end-product formation would scale linearly and proportionately with biomass loading. This was not the case, however. Instead, both total end-product yields and proportional solubilization efficiencies decreased as loadings increased (Table 1). This reduction in solubilization efficiency is similar to observations in free-enzyme systems [16, 18] as well as the CBP-candidate bacterium *C. phytofermentans* [20] under high-solid loading conditions.

The second-round fermentation experiments provided evidence that other factors, in addition to recalcitrance, were limiting end-product formation (Fig. 2). Here, the continued solubilization and end-product formation from the spent switchgrass confirmed that *C. thermocellum*’s hydrolytic machinery was still capable of accessing fermentable substrates in the insoluble portion of the residual biomass. Despite the continued fermentation, however, the ethanol titers achieved during second-round fermentations (Fig. 2) could not fully account for differences in end-product titers observed in the initial switchgrass vs. Avicel comparison experiments (Fig. 1). Multiple possibilities can likely account for these differences. First, a certain proportion of the glucans in switchgrass remained inaccessible to hydrolysis and could not be solubilized. Second, 29–34% of the solubilized and unfermented saccharides recovered in the broths of first-round fermentations were glucans. These saccharides were lost in downstream processing making them unavailable for conversion to end-products. Finally, washing biomass reduces/eliminates fermentation inhibitors as well as readily solubilized sugars [7, 21], but continued hydrolysis may lead to the generation of new inhibitors. Specifically, the second-round fermentations showed decreased solubilization and conversion efficiencies at increased biomass loadings (Fig. 2b) similar to the first-round fermentations.

Given the fermentative capabilities observed in the 17.5 g/L Avicel conditions (Fig. 1c), anabolic limitation due to medium composition or pH-dependent inhibition is considered unlikely explanations for the accumulation of unfermented glucan equivalents in cultures broths. Experiments designed to investigate metabolic inhibition showed robust fermentation by *C. thermocellum* in terms of total soluble sugar utilization (Table 3). Significant changes included the reduction of ethanol and formate titers in the condition containing 50% supernatant from the original 17.5 g/L Avicel fermentation. While there was a 78 and 65% reduction in the net production of ethanol and formate, respectively, compared to the control condition, the titers of these metabolites were actually the highest observed when accounting for end-product carryover from the initial supernatant broths. As the minor changes in substrate utilization cannot account for these differences, these reductions likely represent end-product induced metabolic shifts. End-product-based feedback inhibition has been previously noted in *C. thermocellum* fermentations [29]. In that study, increased ethanol titers were observed to lead to an increase in acetate production. The strain used here, however, is a phosphotransacetylase/lactate dehydrogenase mutant [25], which limits its potential to redirect its metabolites to acetate or lactate. Since significant increases in the metabolites assayed here were not observed (Table 3), it is presumed that metabolic shifts led to increases in amino acids and/or other “overflow” metabolites such as malate, isobutanol, meso-2,3-butanediol, etc. as has been previously reported for growth on model substrates [5, 12, 26]. Production of these metabolites can account for up to 30% of total carbon depending on Avicel loading [12] and likely comprise a large fraction of the undetected carbon in our Avicel mass balances (Table 1) and the end-product induced shifts observed (Table 3, Fig. 3). The effect of these metabolites on *C. thermocellum*’s hydrolysis machinery has not yet been investigated. However, a recent study has shown that the production of “overflow” metabolites is relatively muted during *C. thermocellum* switchgrass fermentations making these compounds unlikely contributors to the switchgrass-derived inhibition observed here [30].

Recently, it has been determined that the non-metabolizable pentose sugar, xylose, can act as a significant electron sink for *C. thermocellum* metabolism [31]. Integrated omics-analyses of *C. thermocellum* switchgrass fermentations have further suggested that significant carbon flux is directed away from glycolytic compounds towards alternative pathways in response to increased solubilized C5 intermediates that accumulate throughout fermentation [30]. While electron loss to non-metabolized lignocellulose-derived compounds may partly explain differences in achievable titers between model and real-world substrates, the extent that this is possible in *C. thermocellum* fermentations requires further investigation. In addition, while non-target electron loss is important to consider in attempts to industrialize ethanol production using *C. thermocellum*, these shifts do not explain why fermentable saccharides remain unfermented in culture broths after 10 days.

Hydrolysis was another process shown to be vulnerable to inhibition. Specifically, at the initial 25 and 50 g/L switchgrass loadings, the solubilized, but unfermented glucose equivalents represent 12 and 11%, respectively, of the initial glucan provided. In those same fermentations, however, the solubilization efficiency decreased by 17 and 26% relative to the 10 g/L loading (Table 2). The data in Fig. 3 further show that the supernatants from the 25 and 50 g/L loadings had the most detrimental effect on Avicel solubilization. All conditions showed significant (*p* < 0.05) inhibition of total Avicel solubilization relative to the control. As cultures containing Avicel-derived supernatants would have no lignocellulose-derived inhibitors, the most plausible explanation is that *C. thermocellum* fermentation products inhibited cellulase activity. Ethanol and other fermentation end-products have been reported to non-competitively inhibit fungal cellulases with concentrations as low as 24 mM (1.09 g/L) ethanol leading to significant reductions in activity [19, 32, 33]. Initial ethanol concentrations here ranged from 6 to 59 mM depending on the source of the spent supernatant, yet absolute titers exceeded 100 mM (4.6 g/L) in all conditions by the end of the hydrolysis experiments. The high titers formed during the course of the experiment may have crossed a threshold concentration, where continued *C. thermocellum* cellulase activity became inhibited. Multiple ethanol tolerant strains have been reported, which in some cases have improved total solubilization capabilities, showing that this barrier can be overcome for *C. thermocellum* [34–37].

The switchgrass-derived supernatants were more inhibitory than those derived from Avicel fermentations (Fig. 3). In these cases, fermentation products alone cannot explain the reduction in solubilization. This is particularly evident in the first-round switchgrass fermentations, where end-product concentrations were significantly lower than those in the Avicel fermentations and did not approach the titers attained on the second-round Avicel fermentations. At the exclusion of *C. thermocellum* metabolites inhibiting hydrolysis, it suggests that the basis for inhibition is derived from the solubilization of switchgrass itself.

Recent studies have shown the adverse effect that lignin has on enzyme accessibility and carbohydrate solubilization in *C. thermocellum* fermentations with technological approaches such as in situ ball milling showing promise to reduce the recalcitrance barrier [38, 39]. Accessibility, however, does not explain the reduced solubilization of Avicel in the spent supernatant experiments observed here. The solubilization of lignin is considered to be quantitatively insignificant in *C. thermocellum* switchgrass fermentations [3], suggesting that the higher inhibition observed in the switchgrass-derived supernatants is not due to lignin-derived compounds.

Other switchgrass-derived components, such as hemicellulose or pectin hydrolysis products, may contribute to hydrolysis inhibition. Soluble xylo-oligomers are known to inhibit cellulase activity [40]. This may be important in high-solid loading fermentations as *C. thermocellum* is known to proportionately solubilize cellulose and hemicellulose fractions equally [3, 9]. In the 50 g/L loadings tested here, xylose equivalents reached concentrations of ~ 5 g/L at the end of 10 days, which is higher than concentrations needed to reduce activity of fungal cellulases [40]. These concentrations, however, reflect measurements performed in homogenized supernatant samples, where diffusion is not limited. In high-solid loadings, mass transfer issues are known to affect oligosaccharide diffusion leading to high localized sugar concentrations [16, 18]. The effects of high localized concentrations may have greater physiological effects on *C. thermocellum* hydrolysis and metabolism than can simply be predicted by determining product concentrations at the end of fermentation.

Fungal cellulases have also been reportedly inhibited by mixed xylo-glucan oligomers [41]. In that study, the inhibitory effect of the oligomers was significantly and differentially reduced after treatment with xylanases, xyloglucanases, or lichenases, suggesting that multiple oligomers contribute in concert to the reduction in cellulase activity observed. Bayer & Lamed [42] have reported that pectin hydrolysis products also reduce the cellulose-hydrolyzing activity of purified *C. thermocellum* cellulosomes. Removal of the low-molecular weight pectin breakdown products restored the hydrolytic activity, however. While the hemicellulose and pectin deconstructing capabilities of *C. thermocellum* have been well documented [3, 9, 43], monoculture environments lack a sink for fermentation of the breakdown products. The catabolism of these products would enable their removal from fermentation broths potentially alleviating the inhibition observed. Additional studies designed to determine the chemical nature and structure of *C. thermocellum* hydrolysis inhibitors are warranted. Such insights could be useful in fully elucidating the inhibitory mechanism(s) and allow the development of new strategies to overcome inhibition.

## Conclusions

Overcoming hurdles related to the recalcitrance barrier, metabolic inhibition and addressing inhibition of hydrolysis are likely required for the industrialization of *C. thermocellum* or other bioconversion strategies for lignocellulosic biofuel production. Efforts to reduce the recalcitrance of minimally pretreated feedstocks are well underway through the use of genetically engineered or natural plant variants that have altered cell wall compositions. For continued improvements to microbial bioconversion, however, the data presented here suggest that inhibition of hydrolysis plays a greater role in reducing biofuel production at higher biomass loadings than does metabolic inhibition. Accordingly, successful efforts to reduce inhibition of hydrolysis may allow significant steps forward in applying CBP with *C. thermocellum *to convert industrially relevant biomass loadings to fuels and chemicals.

## Methods

### Bacterial strains, medium and growth

Lab stocks of *C. thermocellum* M1570 [25] were used throughout this study. Cultures were grown in Medium for Thermophilic Clostridia (MTC) as described [44] with the following exceptions: (i) MOPS buffer was increased from 5 to 10 g/L and (ii) the initial pH of the medium was 7.2–7.4. Switchgrass was milled in a Wiley mill using a 20 mesh screen (Thomas Scientific, Swedesboro, NJ). For the first-round fermentations, Avicel or switchgrass was autoclaved in 25 mL of Milli-Q water (Millipore Corporation, Billerica, MA) under a 100% nitrogen headspace. Preexperiments determined that autoclaving the switchgrass in this manner released 0.55 mM acetate, 0.24 mM acetate, or below detectable amounts of acetate in the 50, 25, or 10 g/L loadings, respectively. Eight molar sodium hydroxide was used to neutralize the acetic acid released in the switchgrass containing bottles, whereas sterile Milli-Q water was added to bottles as necessary to normalize liquid addition across conditions. Twenty-five milliliters of filter-sterilized 2X MTC medium was then added aseptically to each bottle and reiterative cycles of gassing:degassing with 100% nitrogen were performed. Prior to inoculating (10% v/v) the Avicel or switchgrass containing bottles, *C. thermocellum* was grown on 3.5 g/L Avicel for 48 h. All experiments were run for 10 days at 55 °C with orbital shaking at 100 rpm unless otherwise noted.

### Sample processing and fermentation analyses

During first-round fermentations, 1 mL of liquid was removed every 24 h for pH and fermentation end-product analyses. After each sampling, headspace pressure was removed by venting the bottles for 15 s inside an anaerobic chamber filled with an inlet gas of 5% H_2_, 10% CO_2,_ and 85% N_2_. At the end of fermentation, cultures were centrifuged at 8000×*g* for 15 min and the supernatants and pellets analyzed. Fermentation end-products in the supernatant were measured using a Waters Breeze 2 high performance liquid chromatography (HPLC) system (Waters Corp., Milford, MA) equipped with an Aminex HPX-87H column (Bio-Rad Laboratories) and a refractive index detector as described previously [31, 45]. The column temperature was set to 60 °C and the mobile phase was 5 mM H_2_SO_4_ flowing at a rate of 0.6 mL/min. Soluble carbohydrate content was determined via quantitative saccharification assay NREL/TP-510-42618 and HPLC method NREL/TP-510-42623 essentially as described [46] using an Aminex HPX-87P column set to 85 °C. Dry weight measurements of residual substrate/biomass and cell growth were determined by incubating culture pellets at 60 °C until a decrease in weight was no longer observed. Residual solids were then stored at − 20 °C for use in second-round fermentations.

### Second-round fermentations

Residual switchgrass samples from equivalent first-round loading concentrations were pooled together. The solids were washed with ultrapure water at a ratio of 1 L for every 2 g of solids and then again dried at 60 °C until a decrease in weight was no longer observed. The dried, spent switchgrass was then autoclaved in water under an N_2_ headspace and an equal volume of 2X MTC medium was added (as described above). Second-round fermentations were then performed identically to first-round fermentations with the following exceptions: (i) 6 mL cultures were used instead of 50 mL cultures and (ii) samples for end-product analyses were taken only immediately after inoculation and after 10 days of fermentation.

Residual supernatants from the first-round fermentations were combined and neutralized to pH = 7.2 using 8 M NaOH. Milli-Q water was once again used to normalize the liquid addition to the supernatants and maintain a consistent dilution across samples. The neutralized supernatants were sterilized via vacuum filtration through at 0.22 µm filter. For the metabolic inhibition studies, 3 mL of sterilized spent supernatant was combined with 3 mL of filter-sterilized fresh 2X MTC medium containing cellobiose. For the hydrolysis inhibition studies, Avicel was first autoclaved in 1.5 mL of water under an N_2_ headspace. After cooling, 3 mL of spent supernatant plus 1.5 mL of 4X filter-sterilized MTC medium was added to each bottle. All bottles were once again gassed:degassed with N_2_. Inoculum for the cellobiose containing cultures was grown for 24 h in MTC medium containing cellobiose or for 48 h in medium with Avicel as described above. Fermentations were run for 5 days (cellobiose) or 10 days (Avicel) and end-product and mass-balance analyses were conducted at *t* = 0 and at the end of fermentation. To avoid substrate losses for the *t* = 0 measurements, replicate bottles were prepared and sacrificed. The *t* = 0 analyses of the sacrificed cultures were assumed to be equivalent to those allowed to incubate for the duration of the experiment.

For the second-round Avicel experiments, total solubilization was calculated as a function of the residual dry weight measurements plus detectable glucose and cellobiose as measured by HPLC. Values were measured in reference to a control condition that contained water instead of spent supernatant from the initial fermentations and are expressed as a percent reduction in total solubilization observed. Second-round fermentations for both cellobiose and Avicel conditions were run using biological triplicates with whole experiments duplicated (*n* = 6).

## Abbreviations

CBP: consolidated bioprocessing
MTC: medium for thermophilic clostridia
